# SKA1/2/3 is a biomarker of poor prognosis in human hepatocellular carcinoma

**DOI:** 10.3389/fonc.2022.1038925

**Published:** 2022-11-10

**Authors:** Guo-Qiang Song, Tian-Li He, Ke-Jie Ji, Yi-Meng Duan, Jia-Wen Zhang, Guo-Qiang Hu

**Affiliations:** ^1^ Department of Respiratory, Changxing Hospital of Traditional Chinese Medicine, Huzhou, China; ^2^ Department of Radiotherapy, Changxing People’s Hospital, Huzhou, China; ^3^ Department of Cancer Center, Changxing Hospital of Traditional Chinese Medicine, Huzhou, China

**Keywords:** spindle and kinetochore-associated complex subunit (SKA), liver hepatocellular carcinoma, enrichment analysis, biomarker, bioinformatics analysis

## Abstract

**Background:**

Spindle and kinetochore-associated complex subunits 1–3 (SKA1–3) stabilize the kinetochore-attached spindle microtubules in metaphase. Due to the dysregulation in multiple cancers, SKA1–3 is considered a predictor for the prognosis of the patients. However, the potential clinical applications of SKA1–3, particularly in hepatocellular carcinoma (HCC) prognosis and progression, have completely unknown yet.

**Methods:**

For the analysis of SKA1–3 expression and applications in clinics in HCC patients, several databases, such as STRING, UALCAN, GEO, and TCGA, were searched. In addition, the underlying mechanisms of SKA for the regulation of HCC occurrence, development, and progression were also explored.

**Results:**

Compared to the normal controls, HCC patients showed dramatically elevated SKA1–3 expression at the mRNA level, and the values of the area under the curve (AUC) were 0.982, 0.887, and 0.973, respectively. Increased SKA1–3 expression levels were associated with the clinical stage, age, body mass index, tumor grade, tissue subtype, and Tp53 mutation status in HCC patients. The analyses of Kyoto Encyclopedia of Genes and Genome (KEGG) and Gene ontology (GO) demonstrated that SKA1–3 are enriched mainly in the Fanconi anemia, homologous recombination, spliceosome, DNA replication, and cell cycle signaling pathways. The hub genes, such as *CDK1, CCNB1, CCNA2, TOP2A, BUB1, AURKB, CCNB2, BUB1B, NCAPG*, and *KIF11*, were identified in protein–protein interactions (PPIs). The expression levels of hub genes were increased in HCC patients and predictive of a poor prognosis. Finally, the expression levels of SKA1–3 were determined using the GEO database.

**Conclusions:**

SKA1–3 are potential prognostic biomarkers of and targets for HCC. In addition, SKA1–3 may affect HCC prognosis *via* the Fanconi anemia pathway, homologous recombination, spliceosome, DNA replication, and cell cycle signaling pathway.

## Introduction

Liver cancer is the most common cancer worldwide and has high morbidity and mortality rates (1). The patients who were diagnosed as liver cancer worldwide increased from 471,000 in 1990 to 1,007,800 in 2016 (1). Hepatocellular carcinoma (HCC) is considered the most frequently identified primary liver cancer. Patients with similar pathological types may have different underlying molecular etiologies, leading to different responses to treatment (2). The commonly used cancer treatments, such as surgical resection, radiation therapy, chemotherapy, and immune checkpoint therapy, have limited efficacy against HCC (3–6). Therefore, new therapies are required for advanced HCC to improve patient outcomes; in particular, multimodal therapies are required that can improve the tumor response in HCC patients.

Spindle and kinetochore-associated complex subunits (SKAs) stabilize the centromere-attached spindle microtubules during mid-mitosis, which promotes the completion of mitosis. SKAs include SKA1–3 and play important roles in non-neoplastic diseases, such as obsessive-compulsive disorder (4); they also predict the development of post-traumatic stress disorder (5). SKA1–3 affect the occurrence and development of several cancers and is a poor prognosis marker in lung cancer, esophageal carcinoma, and kidney renal papillary cell carcinoma (KIRP) (6).

The SKA1 expression level is increased in non-small cell lung cancer and is associated with cancer progression by regulating cell proliferation, migration, and invasion (7). The SKA2 expression level is upregulated in esophageal carcinoma tissues compared with the adjacent normal tissues. Through enhancing the AKT signaling pathway activity and elevating the expression of certain epithelial–mesenchymal transition-associated markers, such as N-cadherin and Snail, SKA2 could significantly participated in the regulation of esophageal carcinoma cells proliferation, migration, and invasion (8). SKA3 is a target of KIRP and predicts a poor prognosis due to its effects on the RAS/MAPK, PI3K/Akt, hormone estrogen receptor, hormone androgen receptor, DNA damage, and cell cycle (9). Therefore, SKA1–3 are useful prognostic biomarkers and potential therapeutic targets for several cancers.

Previous studies have found that SKA2 accelerates HCC progression *via* Wnt/β-catenin signaling upregulation (10). However, the potential clinical applications of SKA1–3, particularly for HCC prognosis and development, are yet to be fully elucidated. Thus, we performed a comprehensive analysis using several databases and web tools to investigate SKA expression and its relationships with clinical outcomes and prognosis in HCC patients.

## Methods

### The analysis using the cancer genome atlas

As a landmark cancer genomics program for the molecular characterization of more than 33 cancer types, 20,000 matched normal samples and primary cancer were contained in TCGA (http://cancergenome.nih.gov/).

### The analysis using GEPIA and UALCAN

As two opened databases, GEPIA(http://gepia.cancer-pku.cn/) and UALCAN (http://ualcan.path.uab.edu/) are frequently used to analyze the gene expression of the data from TCGA. For the analysis of SKA1–3 and their hub genes expression in HCC and normal samples, the evaluation of the associations of the SKA1–3 with HCC patients’ clinicopathological characteristics (tumor grade, cancer stage, body mass index, age, and stage), and the determination of the SKA1–3’s prognostic values for HCC prognosis, the GEPIA and UALCAN databases were applied.

### The analysis using cBioPortal

To analyze the clinical and genomic data comprehensively, we employed the cBioPortal database (www.cbioportal.org/) to integrates the data sourced from other databases, such as the International Cancer Genome Consortium and TCGA. Meanwhile, we also analyzed and evaluated the SKA1–3 expression at the mRNA level with the cBioPortal database (RNA Seq v2 RSEM).

### The analysis of biological functions

The data for gene expression from 535 HCC patients in HTSeq-fragments per kilobase per million (FPKM) were obtained from TCGA website. The Pearson correlation coefficients (p < 0.001, |r| > 0.4) were applied to screen the co-expressed of genes with SKA1–3. For exploring the potential signal pathways and biological functions mediated by SKA1-3, the package “clusterProfiler” was applied to perform the Kyoto Encyclopedia of Genes and Genome (KEGG) and Gene ontology (GO) analyses of co-expressed genes. The categories of the molecular function (MF), cell composition (CC), and biological process (BP) were contained in the analysis of GO. The data of gene expression from TCGA was analyzed by GSEA. The value of P less than 0.05 was considered as significant difference.

Based on the string database, we constructed a PPI network of the gens co-expressed with SKA. The significant difference was set as the combined score more than 0.7. Then, the Cytoscape 3.6.1 software was applied to import the PPI network. The CytoHubba plug-in was used to screen the hub genes, and the first 10 genes were defined as hub genes.

### The analysis using GEO

The GEO database (https://www.ncbi.nlm.nih.gov/geo/) was created by the National Center for Biotechnology Information in 2000 and contains the data for high-throughput gene expression. GEO is an international public database that collects and organizes microarray data, next-generation sequencing data, and other forms of high-throughput genomic data uploaded by researchers worldwide, including those related to tumors and non-tumor diseases. We searched the GEO database to obtain the relevant data.

### Statistical analysis

The data with normal distribution were showed as means ± standard deviation. The intergroup comparisons were conducted by Student’s t-test. The values at different time points from same individual or group were compared using the paired samples t-test. The data with non-normal distribution were compared by the rank sum test. The P value less than 0.05 was set as the significant difference. All analyses were performed using R 3.6.3(http://www.R-project.org) and R online site(www.xiantao.love/products).

## Results

### SKA1–3 expression in human cancers

**Figure 1:**
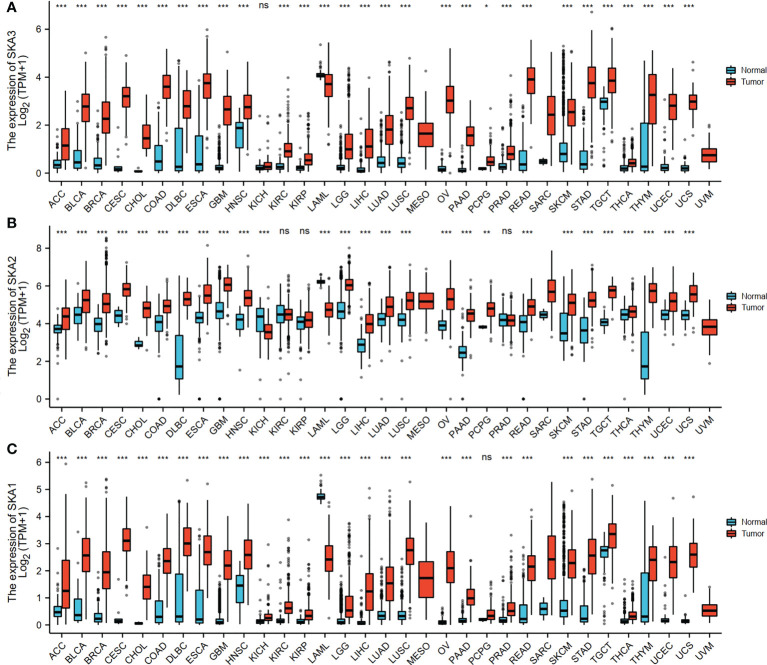
The SKA1/2/3 expression levels in different types of human cancers. **(A)** SKA1, **(B)** SKA2 **(C)** SKA3 (ns, p≥0.05 *P<0.05, **P<0.01, ***P<0.001).

**Figure 2:**
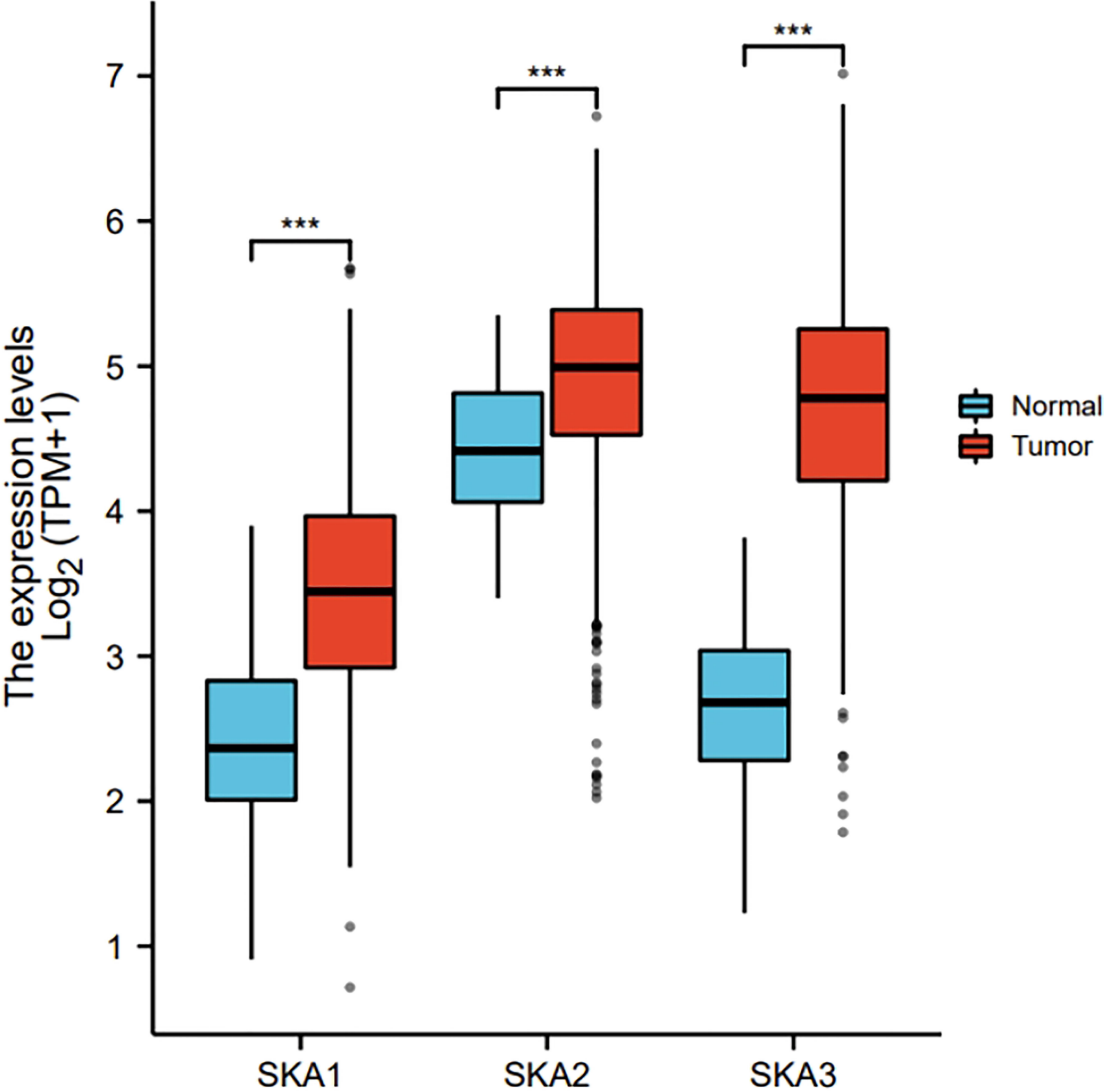
The expression of SKA1/2/3 in HCC in TGCA. SKA, spindle and kinetochore-associated; HCC, Hepatocellular carcinoma; the cancer genome atlas, TGCA (ns, p≥0.05, ***P<0.001).

The RNAseq data with level 3 HTSeq-FPKM format was downloaded from the ALL (Pan-Cancer) project of TCGA (http://cancergenome.nih.gov/). Then, the log2-transformation was performed to the RNAseq data in the FPKM format. SKA1–3 expression between the adjacent normal and tumor tissues is shown in **Figure 1**. Compared with the normal tissues, SKA1 expression was significantly higher in tumor tissues, except in the case of kidney chromophobes. Except for kidney renal clear cell carcinoma, KIRP, and prostate adenocarcinoma, we observed that tumor tissues exhibited significantly high expression of SKA2 compared to that of normal tissues. Additionally, except for pheochromocytoma and paraganglioma, dramatically high expression of SKA3 in tumor tissues was also discovered compared to that in normal tissues. In HCC, SKA1–3 expression levels were all higher in cancer tissues than normal tissues (**Figure 2**).

### SKA1–3 mRNA expression in UALCAN

**Figure 3:**
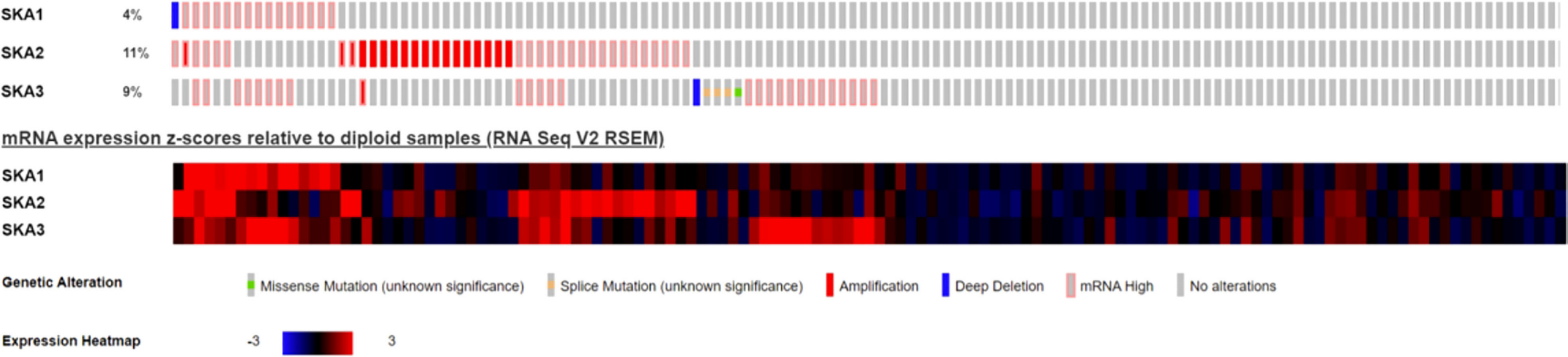
Expression of SKA1/2/3 in cBioPortal database in HCC patients; SKA, spindle and kinetochore-associated; HCC, Hepatocellular carcinoma.

**Figure 4:**
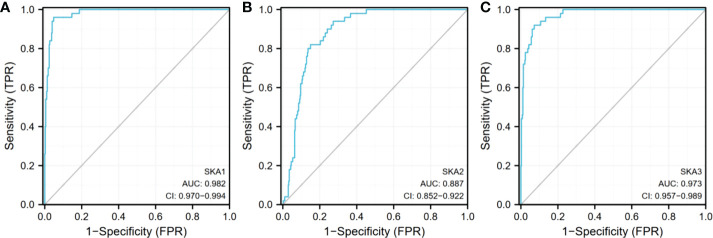
Expression of SKA1/2/3 in TGCA database. **(A)** is SKA1, **(B)** is SKA2, **(C)** is SKA3 SKA1/2/3, spindle and kinetochore associated complex subunit 1/2/3; AUC, area under curve; Cl, confidence level.

**Figure 5:**
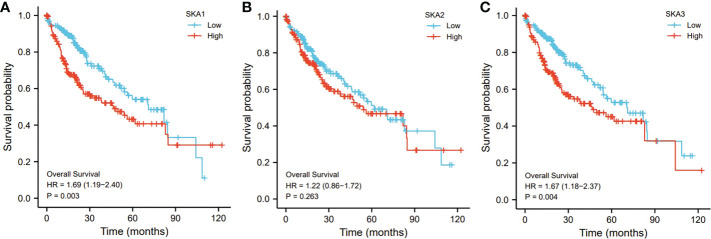
The relationship of SKA1 **(A)**, SKA2 **(B)**, SKA3 **(C)** with OS in HCC patients in TGCA database. SKA, spindle and kinetochore associated; OS, overall survive.

Analysis of the cBioPortal and TCGA databases showed increased frequencies of SKA1–3 mRNA expression in HCC of 4%, 11%, and 9%, respectively (**Figures 3** and **5**), with AUC values of 0.982, 0.887, and 0.973, respectively (p < 0.05; **Figure 4**).

High SKA1–3 levels were closely related to the cancer stage, weight, tumor grade, Tp53 mutation status, tumor histology, and HCC prognosis. The significant correlations of the elevated SKA1–3 expression with the HCC patient’s prognosis and clinicopathological features were observed from the UALCAN database. Additionally, a association of the high SKA1 expression with the tumor histology, Tp53 mutation status, tumor grade, weight, and cancer stage, was also discovered. Meanwhile, we also observed the close relation between the elevated SKA2 expression and the tumor histology, Tp53 mutation status, tumor grade, weight, age, and cancer stage, as well as the correlation between the increased SKA3 expression with the tumor histology, Tp53 mutation status, tumor grade, weight, and cancer stage (p < 0.05; **Table 1**).

**Table 1:** The relationships between SKA1/2/3 expression and clinicopathological features in HCC patients in the UALCAN database.

Clinicopathologic features		SKA1(P value)	SKA2(P value)	SKA3(P value)
Cancer stage				
	Stage1-vs-Stage2	5.64E-03	NS	3.13E-02
	Stage1-vs-Stage3	3.63E-05	3.83E-05	2.62E-04
	Stage1-vs-Stage4	NS	NS	NS
	Stage2-vs-Stage3	NS	3.59E-02	NS
	Stage2-vs-Stage4	NS	NS	NS
	Stage3-vs-Stage4	NS	4.64E-02	NS
Gender				
	Male-vs-Female	NS	NS	NS
Age				
	Age(21-40Yrs)-vs-Age(41-60Yrs)	NS	NS	NS
	Age(21-40Yrs)-vs-Age(61-80Yrs)	NS	NS	NS
	Age(21-40Yrs)-vs-Age(81-100Yrs)	NS	NS	NS
	Age(41-60Yrs)-vs-Age(61-80Yrs)	NS	2.41E-02	NS
	Age(41-60Yrs)-vs-Age(81-100Yrs)	NS	NS	NS
	Age(61-80Yrs)-vs-Age(81-100Yrs)	NS	NS	NS
Weight				
	Normal_Weight-vs-Extreme_Weight	NS	1.10E-02	NS
	Normal_Weight-vs-Obese	NS	NS	3.00E-03
	Normal_Weight-vs-Extreme_Obese	8.58E-03	NS	NS
	Extreme_Weight-vs-Obese	NS	NS	2.43E-02
	Extreme_Weight-vs-Extreme_Obese	NS	NS	NS
	Obese-vs-Extreme_Obese	NS	NS	NS
Tumor grade				
	Grade 1-vs-Grade 2	NS	NS	NS
	Grade 1-vs-Grade 3	6.42E-03	NS	7.63E-04
	Grade 1-vs-Grade 4	4.72E-02	NS	4.52E-02
	Grade 2-vs-Grade 3	4.85E-02	3.41E-04	2.03E-05
	Grade 2-vs-Grade 4	NS	NS	3.49E-02
	Grade 3-vs-Grade 4	NS	NS	NS
Nodal Metastases status				
	N0-vs-N1	NS	NS	NS
Tp53 mutation status				
	TP53-Mutant-vs-TP53-NonMutant	3.81E-08	2.21E-04	2.80E-09
Tumor histology				
	Hepatocellular carcinoma-vs-Fibrolamellar carcinoma	NS	NS	NS
	Hepatocellular carcinoma-vs-Hepatocholangio carcinoma (Mixed)	2.37E-08	NS	7.64E-05
	Fibrolamellar carcinoma-vs-Hepatocholangio carcinoma (Mixed)	NS	NS	7.50E-03

SKA, spindle and kinetochore-associated; NS, no significance.

In addition, compared to those low SKA1- and SKA3-expressed HCC patients, the SKA1- and SKA3-expressed patients exhibited a worse prognosis (p < 0.01; **Figures 5A, C**). However, no significant association of the SKA2 expression with the HCC prognosis was observed (p > 0.05; **Figure 5B**).

And we testify in TGCA database, we found the higher expression of SKA1-3 in tumor group, pathological stage III and IV group, tumor status and AFP more than 400ng/ml group. But there was no significant difference in gender. The expression of SKA2-3 in age less than 60 years was higher than older than 60 years. The expression of SKA1-2 in BMI less than 25Kg/m2 was higher than heavier than 25Kg/m2. The expression of SKA1 and SKA3 in tumor free group were lower than the group with tumor, as well as the patient with vascular invasion (**Table 2**).

**Table 2:** The relationships between SKA1/2/3 with clinicopathological parameters in HCC patients.

Clinicopathological parameters		N	SKA1 expression (2-ΔCq)		SKA2 expression (2-ΔCq)		SKA3 expression (2-ΔCq)	
			Mean ± SD	P value	Mean ± SD	P value	Mean ± SD	P value
Tissue								
	Normal	50	0.074 ± 0.74	0.0001	1.995 ± 0.269	0.001	0.106 ± 0.0106	0.001
	Tumor	373	1.814 ± 1.278		2.807 ± 0.645		1.142 ± 0.751	
Gender								
	Male	253	1.273 ± 0.919	0.471	2.787 ± 0.634	0.305	1.131± 0.767	0.511
	Female	121	1.291 ± 0.796		2.847 ± 0.668		1.164 ± 0.719	
Age(years)								
	<=60	177	1.374 ± 0.928	0.072	2.889 ± 0.672	0.019	1.222 ± 0.783	0.049
	>60	196	1.190 ± 0.829		2.730 ± 0.612		1.066 ± 0.716	
Pathologic stage	I and II	260	1.193 ± 0.820	0.007	2.739 ± 0.626	0.001	1.077 ± 0.695	0.026
	III and IV	90	1.531 ± 0.984		3.008 ± 0.651		1.337 ± 0.866	
BMI	<=25	177	1.393 ± 0.938	0.046	2.893 ± 0.649	0.008	1.203 ± 0.769	0.137
	>25	160	1.180 ± 0.836		2.712 ± 0.630		1.077 ± 0.373	
Tumor status	Tumor free	202	1.154 ± 0.844	0.002	2.763 ± 0.642	0.039	1.030± 0.710	0.002
	With tumor	153	1.429 ± 0.888		2.888 ± 0.652		1.284 ± 0.779	
AFP(ng/ml)	<=400	215	1.102 ± 0.775	0.001	2.725 ± 0.627	0.002	0.967 ± 0.645	0.001
	>400	65	1.600 ± 0.827		2.964 ± 0.576		1.576± 0.712	
Vascular invasion	NO	208	1.087 ± 0.720	0.014	2.756 ± 0.656	0.571	1.024 ± 0.0.693	0.018
	Yes	110	1.387± 0.939		2.805 ± 0.593		1.236 ± 0.766	

SKA, spindle and kinetochore-associated; HCC, Hepatocellular carcinoma; BMI, body mass index.

### Genes co-expressed with SKA1–3

**Figure 6:**
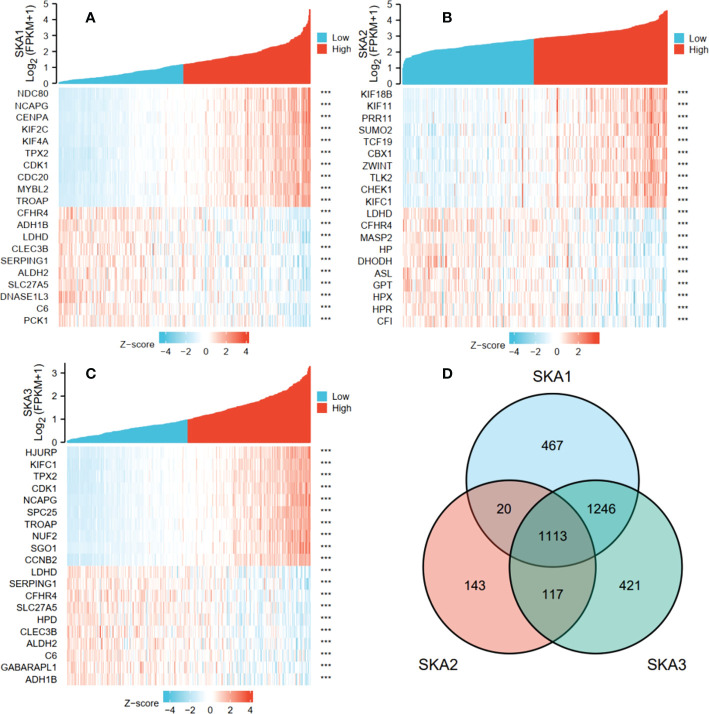
The top 10 genes with positive and negative co-expression of SKA1 **(A)**, SKA2 **(B)**, SKA3 **(C)** in TCGA database according to Heat map and Venn map. **(D)** Intersection co-expression genes of SKA1/2/3. Note: |r| >0.4 and P<0.001. SKA, spindle and kinetochore associated. ***P<0.001.

In TCGA transcriptome data, the numbers of the gene positively correlated with SKA1, SKA2, and SKA3 were 2,757, 1,381, and 2,815, and the numbers of the gene negatively correlated with SKA1, SKA2, and SKA3 were 89, 12, and 82 respectively. **Figures 6A–C** showed the top 10 co-expressed genes correlated with SKA1–3 positively and negatively. Moreover, the 1,113 intersections among SKA1–3 co-expressing genes are shown in a Venn diagram (**Figure 6D**).

### GO and KEGG analyses

**Figure 7:**
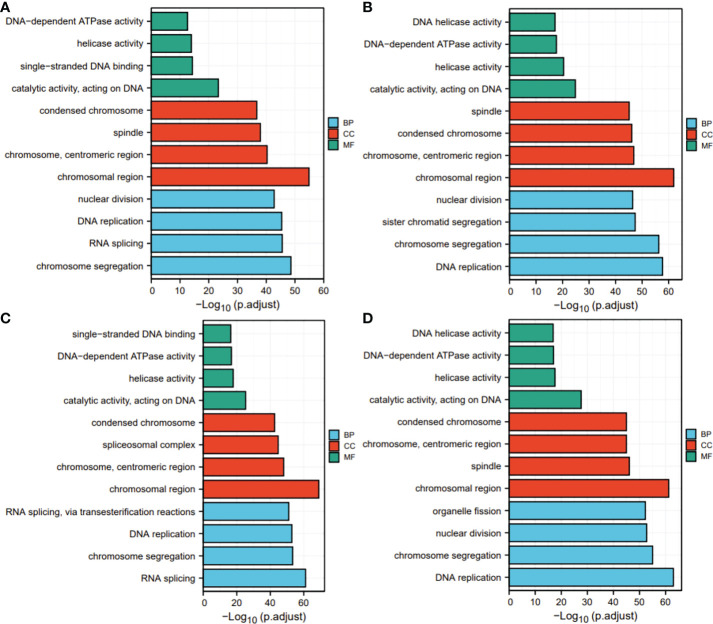
GO analysis of SKA1 **(A)**, SKA2 **(B)**, SKA3 **(C)** co-expression genes, and intersection co-expression genes among with SKA1/2/3 **(D)**. GO, Gene ontology.

**Figure 8:**
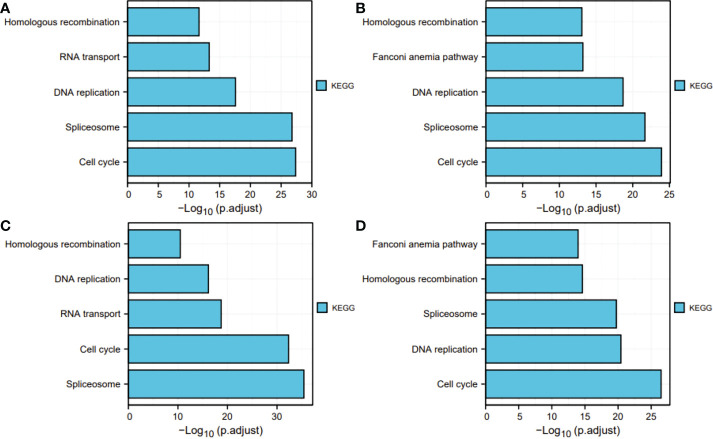
KEGG pathway enrichment analysis of SKA1 **(A)**, SKA2 **(B)**, SKA3 **(C)** co-expression genes, and intersection co-expression genes among with SKA1/2/3 **(D)**. SKA, spindle and kinetochore associated complex subunit. KEGG, Kyoto Encyclopedia of Genes and Genome.

Next, for exploration of the SKA1–3 functions in HCC, we conducted the analyses of GO and KEGG in the genes co-expressed with SKA1–3. The main BPs affected by the SKA1–3 co-expressed genes included DNA replication, chromosome segregation, nuclear division, and organelle fission. The main CCs in associated with the SKA1–3 co-expressed genes included centromeric region, chromosome, condensed chromosome, spindle, and chromosomal region. The main MFs influenced by the SKA1–3 co-expressed genes included DNA-dependent ATPase activity, DNA helicase activity, helicase activity, acting on DNA, and catalytic activity (**Figure 7**). The results from KEGG analysis indicated that the SKA1–3 co-expressed genes regulated the Fanconi anemia pathway, homologous recombination, cell cycle signaling pathway, spliceosome, and DNA replication (**Figure 8**). Our results suggest that in HCCs, SKA1–3 participate in the Fanconi anemia pathway, homologous recombination, spliceosome, DNA replication, and cell cycle signaling pathway.

### Analysis of hub genes in the PPI network

**Figure 9:**
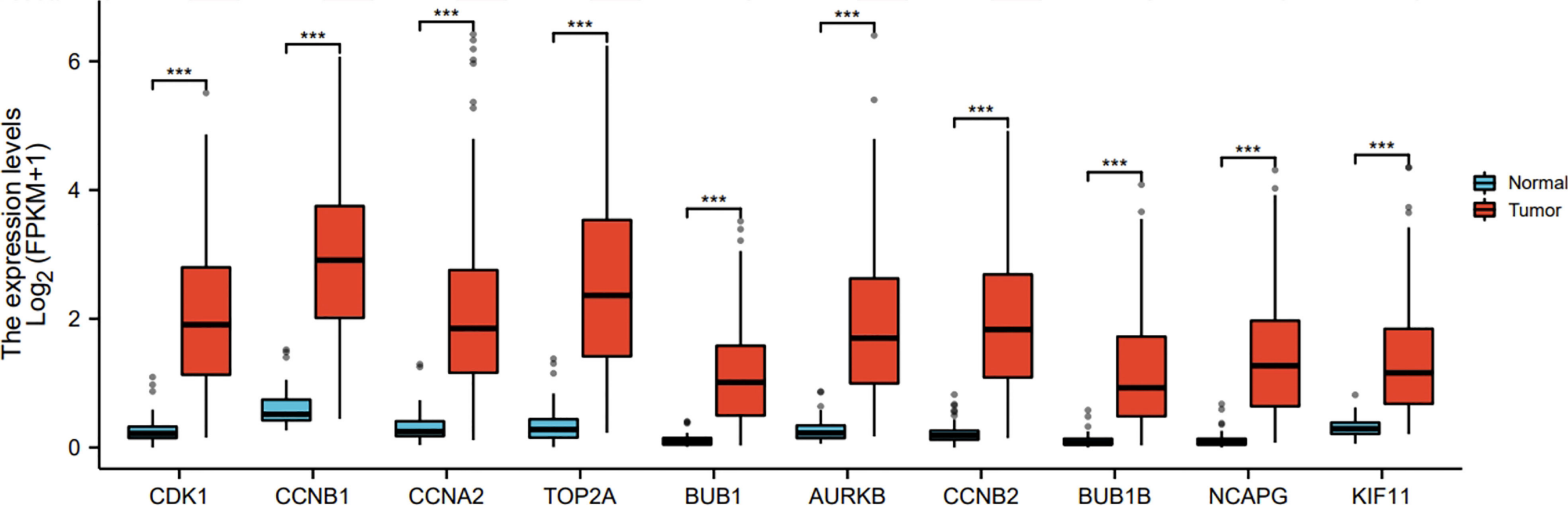
Hub gene expression was increased in HCC in TGCA database. The expressions of CDK1, CCNB1, CCAN2, TOP2A, BUB1, AURKB, CCNB2, BUB1B, NCAPG, and KIF11 were shown. ***P<0.001.

**Figure 10:**
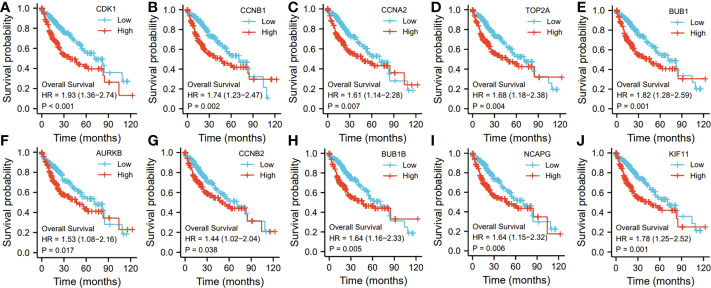
The hub gene related to the overall survival (OS) of HCC patients in TGCA database. The expressions of CDK1 **(A)**. CCNB1 **(B)**, CCAN2 **(C)**, TOP2A **(D)**, BUB1 **(E)**, AURKB **(F)**, CCNB2 **(G)**, BUB1B **(H)**, NCAPG **(I)**, and KIF11 **(J)** were shown.

**Figure 11:**
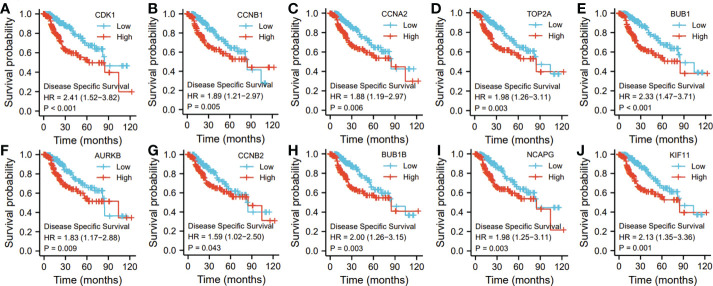
The hub gene related to the disease specific survival (DSF) of HCC patients in TGCA database. The expressions of CDK1 **(A)**. CCNB1 **(B)**, CCAN2 **(C)**. TOP2A **(D)**, BUB1 **(E)**, AURKB **(F)**, CCNB2 **(G)**, BUBIB **(H)**, NCAPG **(I)**, and KIF11 **(J)** were shown.

Ten representative hub genes, such as *CDK1, CCNB1, CCNA2, TOP2A, BUB1, AURKB, CCNB2, BUB1B, NCAPG*, and *KIF11*, were identified using the analysis of PPI network (**Table 3**). TCGA analysis demonstrated that the expression levels of *CDK1, CCNB1, CCNA2, TOP2A, BUB1, AURKB, CCNB2, BUB1B, NCAPG*, and *KIF11* were significantly elevated in the patients with HCC (p < 0.05; **Figure 9**) and associated with the disease-free progression and overall survival (OS) of HCC patients (**Figures 10** and **11**).

**Table 3:** The correlations between the representative 10 Hub genes with SKA1/2/3 in HCC patients.

Rank	Gene symbol	Gene description	Score	SKA1(r)	SKA2(r)	SKA3(r)
1	CDK1	Cyclin-dependent kinases1	206	0.878339	0.608893	0.905994
2	CCNB1	Cyclin B1	175	0.858997	0.512793	0.871675
3	CCNA2	Cyclin A2	173	0.721096	0.506213	0.78519
4	TOP2A	Ubiquitin conjugating enzyme E2C	170	0.821246	0.626492	0.869018
5	BUB1	Budding uninhibited by benzimidazoles 1	164	0.841623	0.578664	0.85081
6	AURKB	Aurora kinase B	159	0.824268	0.549978	0.854956
7	CCNB2	Cyclin B2	157	0.855514	0.605124	0.885941
8	BUB1B	BUB1 mitotic checkpoint serine/threonine kinase B	152	0.829244	0.590812	0.868301
9	NCAPG	(Non-SMC condensin I complex subunit G	152	0.891055	0.575636	0.745865
10	KIF11	kinesin family member 11	152	0.851479	0.663387	0.87353

SKA, spindle and kinetochore-associated; HCC, Hepatocellular carcinoma; r, correlation coefficient.

## External validation

### SKA1–3 mRNA expression levels in the GEO database

**Figure 12:**
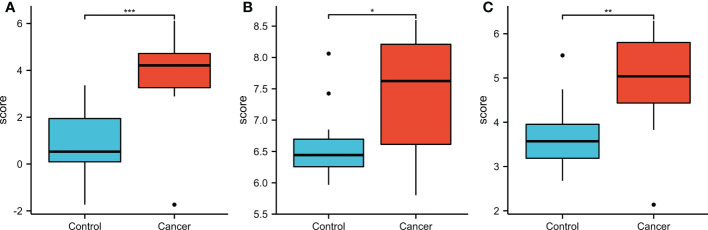
The expression of SKA1 **(A)**, 2 **(B)**, 3 **(C)** in HCC patients in GEO database (GSE 884402). (*P<0.05, **P<0.01, ***P<0.001).

For the validation of the results of SKA1–3 expression, we downloaded GSE84402 (5) datasets from GEO (http://www.ncbi.nlm.nih.gov/geo/) and generated the expression profiling arrays using GPL570 (HG-U133_Plus_2) Affymetrix Human Genome U133 Plus 2.0 Array (Affymetrix, Santa Clara, CA, USA). In addition, the GSE84402 dataset was obtained, which included 28 specimens from 14 paired HCC and corresponding non-cancerous tissues. Similar to TCGA analysis, SKA1–3 expression levels were higher in tumor than normal tissues (**Figure 12**).

## Discussion

As a key component in the motor-binding microtubules (11), SKA aberrantly expression results in spindle checkpoint defects, which play a key role in cell cycle regulation and tumorigenesis (12). Several studies have demonstrated that SKA1–3 are involved in apoptosis and tumorigenesis, and their abnormal expression or activation is common in malignant tumors. As a result, SKAs are significantly correlated with malignant tumors (13).

High SKA1 expression was significantly correlated with tumor size, cellular differentiation, and a poor prognosis in pancreatic ductal adenocarcinoma. In addition, SKA1 in pancreatic ductal adenocarcinoma acts as a tumor promoter, and SKA1 overexpression promoted cell proliferation, migration, and invasion *in vitro* and *in vivo* (14). SKA1 knockdown inhibited cell proliferation, invasion, migration, and cell cycle arrest in human adenoid cystic carcinoma (15). SKA1 was found to be significantly overexpressed in esophageal squamous cell carcinoma cells, and suppression of the SKA1 expression could significantly inhibit the cell cycle progress, cell migration and proliferation, and promote the cell apoptosis (16).

A recent study reported that the mRNA level and immunohistochemical staining of SKA2 were significantly increased in HCC tissues compared with normal tissues (17). Due to the direct targeting of miR-140-3p to SKA2, overexpression of SK2 could partially suppress the inhibitory effect of miR-140-3p restoration in breast cancer cells (18). Another study demonstrated that miR-301, the locus of which is in the intron of the SKA2 gene, is responsible for centromere assembly, and that the two genes are co-expressed in primary breast cancer samples (19). The association of SKA2 with the metastasis of breast cancer was verified by the inhibition of the metastasis and migration of breast cancer observed after the blocking the SKA2 expression, and the underlying mechanism of this association is the E-cadherin translocation from the cytoplasm to the nucleus (20).

SKA3 is significantly particapted in the processes of chromosome cohesion maintenance and silencing of spindle checkpoint during mitosis (21). In the female patients with early breast cancer, the close association of the elevated SKA3 expression with a poor prognosis was observed (22). The inhibition of cell proliferation, induction of cell cycle arrest in the G1/S phase, as well as *in vitro* and *in vivo* tumorigenesis, were significantly connected to the downregulation of SKA3. In addition, SKA3 downregulation led to decreased cyclin D1 expression and retinoblastoma phosphorylation, and increased p21 level, suggesting that SKA3 mediates HCC cell cycle and progression (23). In lung adenocarcinoma, SKA3 promoted metastasis by binding to EGFR and activating the PI3K/AKT signaling pathway (24). The analyses of GSEA and RNA-Seq showed the significant participation of SKA3 in the regulation of the PI3K/Akt signaling pathway and the progression of cell cycle in cervical cancer. Western blot analysis showed that elevated SKA3 expression is significantly and positively associated with the increased expression of p-Rb, E2F1, CDK4, cyclin D1, CDK2, cyclin E2, and p-Akt in HeLa cells (25). In addition, SKA3 knockdown inhibited epithelial–mesenchymal transition (26).

In summary, SKA1–3 expression is correlated with cell apoptosis, proliferation, and cell cycle progression and connected to cancer patients’ poor prognosis. However, the expression patterns and roles of SKA1–3 in HCC are unclear. In the present study, the expression, correlations with clinicopathological features, and underlying mechanisms of SKAs in HCC were explored.

In the present study, SKA1–3 expression was upregulated in HCC patients, which was associated with clinical stage, age, body mass index, tumor grade, tissue subtype, and Tp53 mutation status. Furthermore, increased SKA1–3 mRNA levels were associated with OS (SKA2: hazard ratio = 1.22; 95% confidence interval = 0.86–1.72; p = 0.263). Therefore, SKA1–3 could be used as novel targets of anticancer therapy. The main BPs associated with the genes co-expressed with SKA1–3 included DNA replication, chromosome segregation, nuclear division, and organelle fission. The main CCs in associated with the SKA1–3 co-expressed genes included centromeric region, chromosome, condensed chromosome, spindle, and chromosomal region. The main MFs influenced by the SKA1–3 co-expressed genes included DNA-dependent ATPase activity, DNA helicase activity, helicase activity, acting on DNA, and catalytic activity. The analysis of KEGG demonstrated that the genes co-expressed with SKA1–3 were involved in regulating the Fanconi anemia pathway, homologous recombination, spliceosome, DNA replication, and cell cycle signaling pathway.

Ten representative hub genes, such as *CDK1, CCNB1, CCNA2, TOP2A, BUB1, AURKB, CCNB2, BUB1B, NCAPG*, and *KIF11*, were identified using the analysis of PPI network. The elevated expression of *CDK1, CCNB1, CCNA2, TOP2A, BUB1, AURKB, CCNB2, BUB1B, NCAPG*, and *KIF11* was observed by TCGA analysis in HCC (p < 0.05), and these elevated expressions were significantly connected to the disease-free progression and OS of HCC patients (27). The spindle rotation can be blocked by CDK-1 through the inhibition of the interaction between dynein with LIN-5-ASPM-1 and microtubules at the meiotic spindle poles, while through suppression of the CDK-1, APC could promote the spindle rotation (28). Due to the participation of CCNB1 in the cell cycle of HCC through the regulation of DNA replication, CCNB1 can be used for the diagnosis of early-stage HCC (29). Immunohistochemical analysis showed significantly high level of CCNA2 was observed in the tissues from breast cancer patients compared to that from normal controls (29). By regulating the p-ERK1/2/p-SMAD2/Snail pathway, TOP2A could enhanced the process of epithelial–mesenchymal transition and subsequently promote the metastasis of HCC (27). In addition, the HCC patients with the invasion into vascular, dramatically elevated BUB1B and CCNB1 expressions were observed (*p* < 0.05 for both) (30). Additionally, in comparison to the normal controls, the sarcoma tissues and cells exhibited obviously high BUB1, BUB1B, and BUB3 expression (31). High AURKB expression was significantly related to poor prognosis in neuroblastoma patients (32). AURKB knockdown promotes apoptosis in carboplatin-resistant cells *in vitro* (32). Knockdown of CCNB2 mRNA could inhibit cell proliferation, reduce breast cancer cell migration, block the G2/M cell cycle transition, and increase cell apoptosis (33). NCAPG silencing inhibited cell proliferation and induced apoptosis in endometrial cancer cells *via* inhibiting the Wnt/β-catenin pathway (34). Because of the depletion of KIF11 could suppress the growth of cells and tumors *in vitro* and *in vivo*, it’s believed that KIF1I could predict the prognosis of HCC (35)

We examined tissue samples from patients using the GEO database and found that SKA1–3 mRNA levels were higher in HCC tissues than in adjacent tissues. However, we did not perform *in vitro* or *in vivo* experiments. The potential application of SKA1-3 for the development and pathogenesis of HCC, as well as the related oncogenic signaling pathways were analyzed in our present study to develop multi-targeted and SKA1–3-targeted therapies.

Our study had some limitations. First, we used only the GEO database to validate our findings. Second, additional HCC samples are required to determine the interactions between SKAs and the mechanisms regulating HCC development and progression. Third, further research is needed to identify SKA functions at the cellular level.

SKA1–3 mRNA levels were dramatically increased in HCC samples and were significantly correlated with the clinical stage, age, body mass index, tumor grade, tissue subtype, Tp53 mutation status, and prognosis of HCC patients. SKA1–3 can be used to predict the prognosis of HCC patients and treat HCC. Our results will provide novel insight underlying the molecular mechanism of HCC and new therapeutic strategies related to SKA regulation. However, the verification of our present findings and development of clinical applications of SKA as promising treatment targets and prognostic biomarkers should be done in further using experimental studies.

## Data availability statement

The original contributions presented in the study are included in the article/Supplementary Material. Further inquiries can be directed to the corresponding author.

## Author contributions

G-QS write this paper. T-LH polish this paper. K-JJ analysis all data. Y-MD check grammar. J-WZ check the tables and figures. G-QH design this paper. All authors contributed to the article and approved the submitted version.

## Conflict of interest

The authors declare that the research was conducted in the absence of any commercial or financial relationships that could be construed as a potential conflict of interest.

## Publisher’s note

All claims expressed in this article are solely those of the authors and do not necessarily represent those of their affiliated organizations, or those of the publisher, the editors and the reviewers. Any product that may be evaluated in this article, or claim that may be made by its manufacturer, is not guaranteed or endorsed by the publisher.
